# Identifying Themes for an Initial Beta Version of a Mobile Health App for Latino and Native Hawaiian/Pacific Islander Communities: Co-Design and Community-Based Participatory Research in a Code to Community Study

**DOI:** 10.2196/76178

**Published:** 2025-06-12

**Authors:** Christina K Holub, Amy L Barrera, Rosalva Romero Gonzalez, Diane Hoang, Luna Prieto, Samuelu Fesili, Tiana Smith, Harleen Kaur, Cassandra Surban, Michael Markidis, Tana Lepule, Konane Martinez

**Affiliations:** 1 Department of Public Health California State University, San Marcos San Marcos, CA United States; 2 National Latino Research Center California State University, San Marcos San Marcos, CA United States; 3 Pacific Islander Collective San Diego San Diego, CA United States

**Keywords:** community co-design, community-based participatory research, CBPR, mobile health, mHealth

## Abstract

Through a co-design and community-based participatory approach with Latino and Native Hawaiian/Pacific Islander communities, we identified key themes for an initial beta version of a mobile health app, including priorities regarding mental health, access to resources, and chronic disease prevention. Social and cultural connectedness and generational approaches emerged as important strategies for successful intervention design and long-term adoption.

## Introduction

Adopting innovations has become increasingly important, driven by postpandemic growth in the use of technology for wellness and health care [1]. New social norms, attitudes, and behavior patterns impact health and participation in health behavior interventions. Latino and Native Hawaiian/Pacific Islander (NHPI) populations, among the fastest-growing minority US populations [2], experience exacerbated health disparities. Heart disease, cancer, and diabetes are leading causes of death in these populations, and both have high rates of obesity [3]. Latino and NHPI populations are less likely to receive mental health services [3], even though stigmas have decreased [4].

Formative research is essential to informing culturally tailored and innovative strategies to reduce health disparities and address community priorities. Preliminary analysis of PIC Health (Pacific Islander Community Health/Elige Salud) study data will inform the first phase of developing a beta version of a mobile health (mHealth) app. This will enable collection of detailed feedback on the app’s acceptability, feasibility, and general design from community leaders, members, and other stakeholders. This study aims to identify community priorities and general themes to build the mHealth beta app.

## Methods

Through a co-design and community-based participatory research (CBPR) approach, we partnered with Latino and NHPI communities to conduct 12 key informant interviews, 4 focus groups, and 2 community stakeholder meetings, divided equally across the groups. Participants were recruited by convenience sampling at cultural organizations, clubs, and community events. Latino-identifying participants (8 female, 3 male) were primarily of Mexican origin and NHPI participants (8 female, 7 male) included Samoan, Native Hawaiian, and Chamorro people. All participants resided in North County, San Diego, and were older than 18 years (range: 18-69 years).

Key informant interviews and focus groups were recorded, transcribed, and uploaded into ATLAS.ti for qualitative coding and analysis. At least two research team members coded, verified, and extracted themes from the transcripts, which were analyzed using directed content analysis to identify and categorize meaningful information about participants’ opinions, perceptions, and experiences [5]. Coding strategies included the selection of responses for emergent themes and a priori categories [6]. Emergent themes were verified through team discussions and the coding framework. Once themes were identified, the research team worked with a senior software engineer and computer science students to design a progressive web application (PWA; a website that behaves like a mobile app) as an initial beta mHealth app. The research was deemed exempt by the California State University, San Marcos institutional review board, with informed consent waived (1858351). Data were anonymized and participants received US $30 gift cards for interviews and US $20 gift cards for focus groups.

## Results

Across all participants, three major themes were identified for community health priorities: (1) mental health, (2) access to resources and health education, and (3) attention to chronic disease prevention (eg, diabetes, cancer, heart disease). Latino participants emphasized access to health resources and NHPI participants emphasized mental health. In response to questions about improving intervention success, both communities identified two major themes: (1) social and cultural connectedness and (2) attention to generational approaches (Table 1). For the latter, Latino participants identified a need for intergenerational strategies (across generations), while some NHPI participants mentioned multigenerational strategies (tailoring to specific generations).

**Figure 1:**
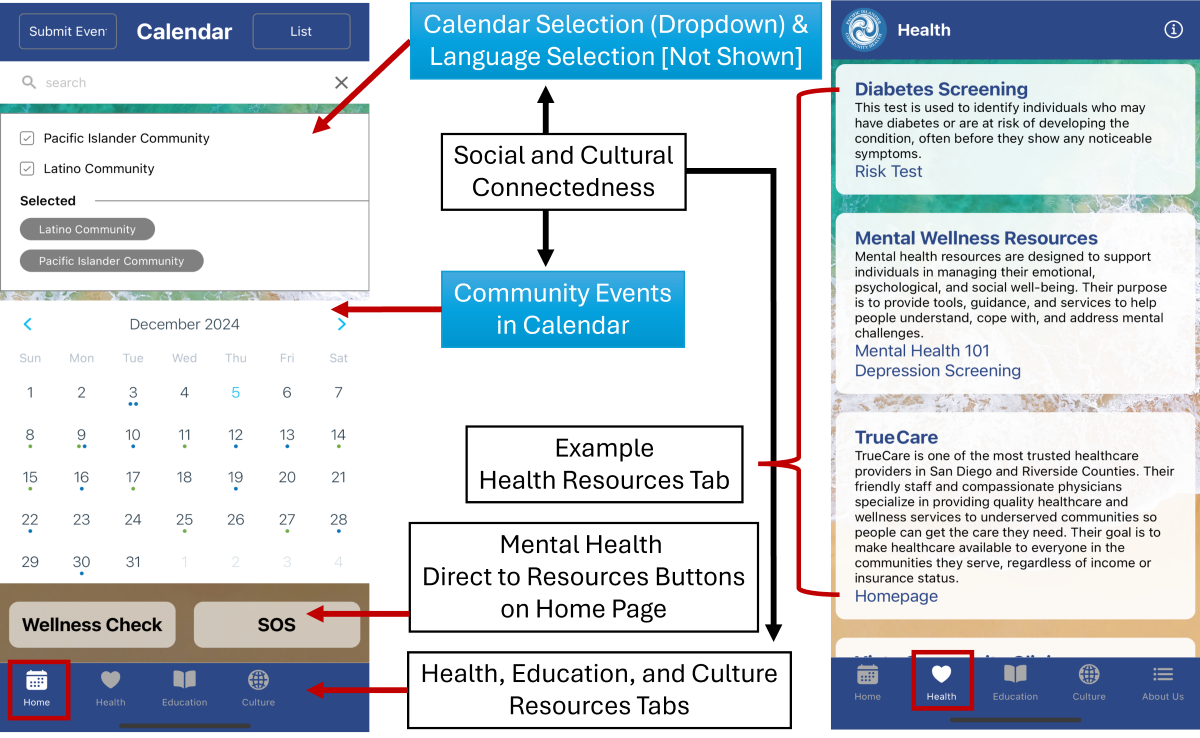
Mobile app theme alignment and components, including social connectedness (community calendar), cultural connection (app design, language, and culturally tailored resources), and community resources (separate tabs for health, education, and a combined resource section for culture, arts, and language).

Based on community stakeholder meetings, theme prioritization, and adaptability to different cultures, the beta mHealth app was created in the PWA format. Figure 1 depicts the alignment between key themes and their corresponding app components.

**Table 1:** Themes, definitions, and participant quote examples.

Theme		Definition; participant describes...	Example
**Community priorities**			
	Mental health	Psychological and emotional well-being; mental health	“[W]hen I’m thinking about the young people that we’ve worked with is, um, mental health and access to mental health.” [NHPI^a^ participant] “Also, I feel like there’s just like a stigma with like mental health, you know, sometimes with the community, with like behavioral health and stuff like that...” [Latino participant]
	Access to health resources	Need for access to health services or options to improve approaches to health services; not only the first access but also continuous service	“If we just had access to education or if we just had better trust with some of these things – [family member] would not have passed away.” [NHPI participant] “It’s still a lack of access. Just a lack of knowledge of what, like, what services are out there.” [Latino participant]
	Chronic disease prevention	Diseases or conditions like hypertension, diabetes, obesity, and other chronic diseases; could be mentioned as underlying conditions	“I see a lot of diabetes related complications.” [Latino participant] “I would say they struggle with like obesity and diabetes and gout heart disease are the big ones.” [NHPI participant]
**Intervention success**			
	Social and cultural connectedness	Specific ways to connect to culture; cultural protocols	“Definitely reminding people, at least on the Latino side for me with my family, like that connection back to a little more of like our roots and more like, because real, traditional, healthy, organic Mexican food, it’s not bad. It’s all the additional everything that we end up with, particularly in the US.” [Latino participant] “I love it when I see...programs where they can connect to traditions.... You know this is what our ancestors did, and this is why they did it, or this was their philosophy, like I love it when they can take something that’s you know...contemporary. You know, but still connecting it to the past.” [NHPI participant]
**Generational Approaches**			
	Intergenerational and multigenerational	Approaches for the different age groups or generations	“And I think I'd agree with that really having the younger generation, you know…They're the ones that are being exposed to…nutrition classes in school… and really encouraging students to go home and share this with your parents. Go home and share this with…mom and dad or grandma and grandpa.” [Latino participant] “…If you had exclusively for that age group meeting. I don't think putting them all together in a forum would be the ideal.” [NHPI participant]

^a^Native Hawaiian/Pacific Islander.

## Discussion

Our research identified major themes from qualitative data that translated into components of an initial mHealth app beta designed for the Latino and NHPI communities. The themes identified in the preliminary analysis for community health priorities include mental health, access to resources, and chronic disease prevention. Themes surrounding intervention success include social and cultural connectedness and generational approaches. Many themes overlap between the Latino and NHPI communities, but cultural details and tailoring should differ.

The initial beta incorporates both communities; however, future work may lead to the need for separate mHealth apps or other advanced strategies to tailor information in real time, such as the use of artificial intelligence to populate appropriate resources, although challenges related to artificial intelligence in mHealth must be addressed [7]. The next phase of app development will include gathering additional community insights on usability, acceptability, and design improvements through a mixed methods approach. Feedback and ongoing co-design collaboration in this next phase will result in a final mHealth app for beta testing. Overall, to address health equity, co-design and CBPR approaches with the communities who benefit are essential for adoption of new technologies and greater mHealth intervention success.

## Abbreviations

CBPR: community-based participatory research
DCA: directed content analysis
mHealth: mobile health
NHPI: Native Hawaiian/Pacific Islander
PIC Health: Pacific Islander Community Health (Elige Salud)
PWA: progressive web application
