# Correction: Identifying Themes for an Initial Beta Version of a Mobile Health App for Latino and Native Hawaiian/Pacific Islander Communities: Co-Design and Community-Based Participatory Research in a Code to Community Study

**DOI:** 10.2196/79194

**Published:** 2025-07-02

**Authors:** Christina K Holub, Amy L Barrera, Rosalva Romero Gonzalez, Diane Hoang, Luna Prieto, Samuelu Fesili, Tiana Smith, Harleen Kaur, Cassandra Surban, Michael Markidis, Tana Lepule, Konane Martinez

**Affiliations:** 1 Department of Public Health California State University, San Marcos San Marcos, CA United States; 2 National Latino Research Center California State University, San Marcos San Marcos, CA United States; 3 Pacific Islander Collective San Diego San Diego, CA United States

In “Identifying Themes for an Initial Beta Version of a Mobile Health App for Latino and Native Hawaiian/Pacific Islander Communities: Co-Design and Community-Based Participatory Research in a Code to Community Study” (JMIR Form Res 2025;9:e76178), the authors note one omitted row in Table 1.

Table 1 has been revised to include the theme “generational approaches”, its definition, and participant quotes as the last row.

Below is the added row with its definition and participant quotes:

**Table 1:** Themes, definitions, and participant quote examples (added row).

Theme		Definition; participant describes...	Example
**Generational Approaches**			
	Intergenerational and multigenerational	Approaches for the different age groups or generations	“And I think I'd agree with that really having the younger generation, you know…They're the ones that are being exposed to…nutrition classes in school… and really encouraging students to go home and share this with your parents. Go home and share this with…mom and dad or grandma and grandpa.” [Latino participant] “…If you had exclusively for that age group meeting. I don't think putting them all together in a forum would be the ideal.” [NHPI participant]

The correction will appear in the online version of the paper on the JMIR Publications website, together with the publication of this correction notice. Because this was made after submission to PubMed, PubMed Central, and other full-text repositories, the corrected article has also been resubmitted to those repositories.

